# Prevalence and factors associated with obesity in Rwanda: Evidence from the 2022 NCD STEPS Survey

**DOI:** 10.1371/journal.pgph.0004736

**Published:** 2026-04-10

**Authors:** Nshimiyimana Gad, Bakang Percy Tlhaloganyang, Jean Claude Habineza, Niyonsenga Simon Pierre, Clarisse Musanabaganwa, Eric Remera, Amparo Elena Gordillo-Tobar, Alberto Barcelo, Fabrice Iradukunda, Ukuri Sincere Josue, Ntaganda Evariste, Akandi Pilote, Igiraneza Pacifique, Ndagijimana Albert, Uwinkindi Francois, Muvunyi Mambo Claude

**Affiliations:** 1 Rwanda Biomedical Centre, Kigali, Rwanda; 2 World Bank Group, Washington, District of Columbia, United States of America; 3 Department of Public Health Sciences Research, University of Miami, Miami, Florida, United States of America; 4 School of Public Health, College of Medicine and Health Sciences, University of Rwanda, Kigali, Rwanda; Universiti Malaya, MALAYSIA

## Abstract

Obesity is an emerging public health concern in Rwanda, yet nationally representative data on its associated factors remain limited. This study analyzes recent survey data to assess the prevalence of obesity and explore associated demographic, behavioral, and metabolic characteristics. The study analyzed data from the 2022 Rwanda NCD STEPS Survey, a nationally representative cross-sectional study of 5,509 adults aged 18–69 years. Data collection followed the WHO STEPwise approach and included structured questionnaires, physical measurements, and biochemical tests. Obesity was defined as a body mass index (BMI) ≥30 kg/m^2^. Poisson regression with robust standard errors was used to estimate crude, age- and sex-adjusted, and fully adjusted prevalence ratios (PRs) with 95% confidence intervals (CI) to describe associations between obesity and selected characteristics. The prevalence of obesity in the study population was 4.3% (95% CI: 3.7–5.1%). Obesity was significantly more common among women (APR: 4.2, 95% CI: 2.1–8.5) and adults aged 60–69 years (APR: 2.7, 95% CI: 1.03–6.9) compared to younger adults. Rural residents had a substantially lower prevalence of obesity than urban residents (APR = 0.30, 95% CI: 0.20–0.50). Individuals with primary education (APR: 1.7, 95% CI: 1.02–3.0) were more likely to be obese, while individuals earning less than USD 33/month had lower obesity prevalence (APR: 0.5, 95% CI: 0.2–0.9). Past drinkers (APR: 0.5, 95% CI: 0.3–0.9) and non-heavy drinkers (APR: 0.5, 95% CI: 0.3–0.9) had lower obesity prevalence compared to non-drinkers. Hypertension remained significantly associated with obesity (APR: 1.7, 95% CI: 1.1–2.5). Obesity in Rwanda was significantly associated with sex, age, residence, education, income, alcohol use, and hypertension. Interventions should prioritize older women in urban areas and integrate education-sensitive strategies, alcohol-related risk reduction, and hypertension management into obesity prevention programs.

## Introduction

Overweight and obesity are recognized as major public health threats globally due to their strong association with non-communicable diseases (NCDs), including cardiovascular disease, diabetes, and certain cancers [1,2]. These conditions result from excessive fat accumulation that may impair health and are influenced by a range of behavioral, environmental, and metabolic factors [2,3]. The rising prevalence of obesity is not only a concern in high-income countries but is increasingly affecting low- and middle-income countries (LMICs), particularly in sub-Saharan Africa, due to ongoing nutrition and lifestyle transitions [3,4]. Rwanda, like many LMICs, is experiencing this double burden of malnutrition, where persistent undernutrition now coexists with increasing rates of overweight and obesity. This trend has been observed across different age groups, including adolescents and adults [5–7].

Obesity is often associated with modifiable behaviors, including low levels of physical activity, frequent snacking, and consumption of high-fat and high-sugar foods [3,8]. A policy landscape analysis in Rwanda revealed limited implementation of preventive strategies targeting obesity, particularly regarding the marketing and consumption of sugar-sweetened beverages [8,9]. Despite these growing concerns, much of the evidence on obesity in Rwanda remains fragmented, with most studies focusing on adolescents or urban populations and relying on sub-national data [10,11].

There is a clear need for nationally representative data to better understand the current burden of obesity and its associated risk factors in Rwanda. While the 2022 Rwanda NCD STEPS Survey reported descriptive national estimates, no peer-reviewed analysis has yet examined the independent sociodemographic, behavioral, and metabolic correlates of obesity using this dataset. This study therefore provides the first adjusted analysis of the 2022 STEPS microdata, offering new insights into population-level predictors of obesity in Rwanda to inform evidence-based prevention and control strategies.

## Methods

### Ethics statement

Ethical approval for the 2022 Rwanda Non-Communicable Disease (NCD) STEPS Survey was obtained from the Rwanda National Ethics Committee (RNEC), Ministry of Health, Rwanda (Approval No. 533/RNEC/2021; approved on 18 May 2021) [12]. Administrative clearance was also granted by the Rwanda Ministry of Health.

Written informed consent was obtained from all participants prior to enrollment in the survey. Participation was voluntary, and respondents were informed of their right to withdraw at any time without consequences. To ensure confidentiality, all data were anonymized using unique identifiers, and no personal identifying information was included in the analytical dataset.

### Study design

This study analyzed data from the 2022 Rwanda Non-Communicable Disease (NCD) STEPS Survey, a nationally representative, cross-sectional survey conducted from October 2021 to January 2022, targeting adults aged 18–69 years. The survey adhered to the World Health Organization’s STEPwise approach to surveillance (STEPS) and was coordinated by the Rwanda Biomedical Centre (RBC) and the Ministry of Health [12].

**Fig 1:**
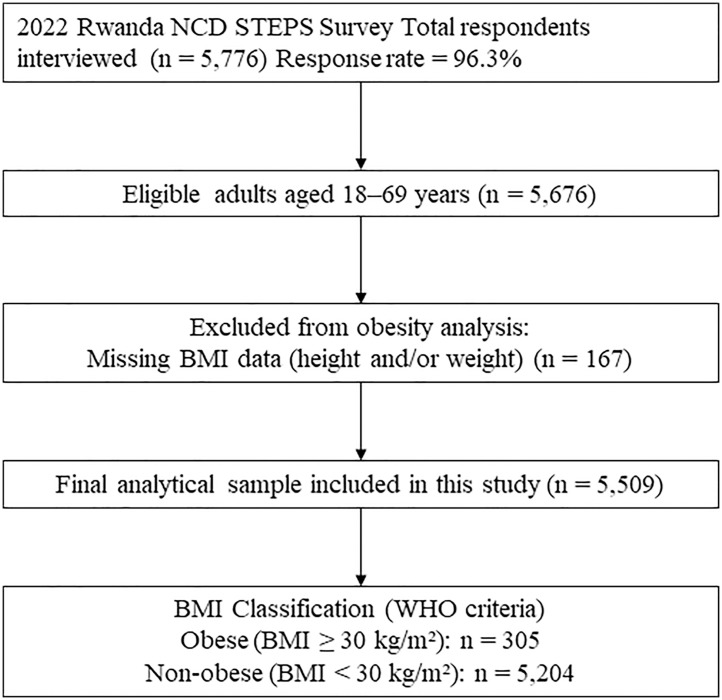
Flowchart of participant selection.

A three-stage stratified cluster sampling strategy was used to ensure national representativeness. In the first stage, 400 enumeration areas (EAs) were selected from all 30 districts using probability proportional to size, with 280 EAs allocated to rural areas and 120 to urban areas. In the second stage, 15 households were systematically selected per EA. In the third stage, one eligible adult aged 18–69 was randomly selected from each household using the STEPS app. This yielded a final sample of 5,676 respondents, with a response rate of 96.3% [12]. The participant selection process for this secondary analysis is illustrated in Fig 1.

### Data collection

Data were collected through face-to-face interviews using electronic tablets preloaded with the eSTEPS data collection application, a digital tool with automated skip patterns, internal consistency checks, and real-time validation to improve data quality and minimize entry errors. The tools were translated into Kinyarwanda and French and pilot-tested to ensure cultural and linguistic appropriateness. Interviews were conducted in private settings to encourage honest responses. Additionally, potential recall and social desirability bias related to self-reported behaviors were mitigated through standardized questioning, interviewer intensive training, and consistency checks within the electronic data collection system. All instruments used for physical and biochemical measurements were standardized and regularly calibrated. The survey team included supervisors and field coordinators for monitoring and routine quality control checks during fieldwork.

Ethical approval for the 2022 Rwanda NCD STEPS Survey was granted by the Rwanda National Ethics Committee under reference number 553/RNEC/2021, dated 18 May 2021. Administrative clearance was also obtained from the Ministry of Health. Written informed consent was obtained from each participant prior to enrollment in the study. To protect confidentiality, all data were anonymized using unique participant codes, and no personal identifiers were retained in the final dataset. Participants with abnormal results (e.g., high blood pressure or elevated glucose levels) were referred to the nearest health facility according to national clinical referral guidelines [12].

### Measurements and definitions

The 2022 Rwanda NCD STEPS Survey was comprised by three components: questionnaire-based data collection, physical measurements, and biochemical assessments. Sociodemographic information and behavioral risk factors such as tobacco use, alcohol consumption, diet, and physical activity were collected through interviewer-administered questionnaires administered in participant’s preferred language.

Anthropometric measurements were collected using standardized protocols. Weight was measured in kilograms using a pre-calibrated digital scale, and height was measured in centimeters using an ultrasonic stadiometer. Waist and hip circumference were measured using a flexible measuring tape. Blood pressure was measured using OMRON automatic digital monitors. Each participant was asked to rest for 15 minutes before the measurement, and three readings were taken at three-minute intervals. The mean of the readings was used for analysis.

Biochemical measurements were conducted using CardioChek PA devices, which were used to assess fasting blood glucose and lipid profiles. Participants were instructed to fast overnight prior to the test [12].

Hypertension was defined as having a systolic blood pressure of at least 140 mmHg, a diastolic pressure of at least 90 mmHg, or being prescribed antihypertensive medication. Diabetes was defined as a fasting blood glucose level of 126 mg/dL or higher, or current use of prescribed antidiabetic medication [12].

### Outcome variable

The primary outcome variable in this study was obesity. It was derived from measured anthropometric data and defined as a binary variable, where individuals with a body mass index (BMI) equal to or greater than 30.0 kg/m^2^ were categorized as obese, and those with a BMI below 30.0 kg/m^2^ were categorized as not obese. BMI was calculated using the standard formula: weight in kilograms divided by height in meters squared (kg/m^2^). This classification was based on internationally accepted WHO criteria and was applied uniformly across all respondents included in the analysis [12].

### Independent variables

The study examined a range of independent variables categorized into demographic, behavioral, and biological domains. Demographic variables included sex (male or female), age group (categorized in 18–29, 30–44, 45–59, 60–69 year intervals), marital status (Single, married or Separated/Widowed/Divorced), education level (no formal education, primary, secondary or higher), place of residence (urban or rural), and household wealth status based on Rwanda’s Ubudehe categorization system.

Behavioral variables were derived from Step 1 of the STEPS survey and included current tobacco use (yes or no), current alcohol consumption (yes or no), fruit and vegetable intake (categorized based on required 5 servings daily), and physical inactivity (categorized based on if or not the participants met the required 600 MET-minutes/week), measured using the Global Physical Activity Questionnaire module embedded in the STEPS tool. Dietary behavior regarding the frequency of consumption of processed foods was also considered.

Biological variables included hypertension and diabetes status, both of which were defined using standard thresholds. Hypertension was based on measured blood pressure values or current antihypertensive medication use, while diabetes was based on fasting blood glucose levels or self-reported use of diabetes medication. These variables were selected based on prior literature and biological plausibility as factors potentially associated with obesity [12].

### Statistical analysis

All statistical analyses were performed using Stata version 17.0, incorporating survey weights to account for the complex sampling design. Survey weights were calculated based on the sampling probabilities at each stage and adjusted for non-response, ensuring that the results are nationally representative of the adult population in Rwanda aged 18–69 years.

Descriptive statistics were used to estimate the weighted prevalence of obesity and the corresponding 95% confidence intervals (CIs) across key demographic, behavioral, and biological characteristics. To assess associations between obesity and explanatory variables, Poisson regression models with robust standard errors were used to estimate crude, age- and sex-adjusted, and fully adjusted prevalence ratios (PRs). Independent variables included in the multivariable model are the ones with p-value <0.05.

Variables included in the multivariable model were selected based on statistical significance in univariate analyses (p < 0.05), in addition to age and sex, which were included a priori due to their established relevance in obesity research. Before model fitting, multicollinearity among independent variables was assessed using variance inflation factors (VIFs), and no concerning correlations were detected (all VIF values < 5). Observations with missing data on key variables were excluded through complete-case analysis, as the proportion of missingness was minimal (<5%).

All categorical variables were modeled with appropriate reference groups, allowing for estimation of relative prevalence across categories without the need for explicit exposed or unexposed labels. The outcome variable, obesity, was treated as binary, coded 1 for individuals with a BMI ≥ 30.0 kg/m^2^ and 0 otherwise.

## Results

The study analyzed a weighted sample of 5,509 adults, among whom 305 were classified as obese based on BMI measurements. The weighted baseline prevalence of obesity was 4.3% (95% CI: 3.7%–5.1%). The mean age was 34.6 years for males and 35.8 years for females. Males had an average weight of 60.4 kg, a mean height of 167.2 cm, and a mean BMI of 21.6 kg/m^2^. In contrast, females had an average weight of 58.8 kg, a mean height of 159.0 cm, and a higher mean BMI of 23.3 kg/m^2^.

Analysis of obesity prevalence across key demographic predictors given in Table 1 showed several important patterns. Women exhibited a significantly higher prevalence of obesity (7.4%) compared to men (1.4%), highlighting the gender disparity in obesity rates in Rwanda. Obesity prevalence increased with age, with the highest prevalence observed among individuals aged 60–69 years (6.0%), while the youngest age group (18–29 years) had the lowest prevalence (2.6%). Marital status was also associated with obesity prevalence, with separated, widowed, or divorced individuals recording the highest prevalence (6.3%), followed by married individuals (5.5%) and single individuals (1.7%). Urban residents had a much higher prevalence of obesity (11.6%) compared to rural residents (2.7%), reflecting the potential influence of urban living environments on obesity patterns.

**Table 1:** Distribution of obesity prevalence by demographic, socioeconomic, behavioral and health-related characteristics.

Characteristics	Frequencies	Obesity, % (95% CI)	
		Yes (n = 305)	No (n = 5204)
**Demographics Characteristics**			
**Gender**			
Male	2126	1.4 (0.9-2.1)	98.6 (98.0 - 99.0)
Female	3383	7.4 (6.4-8.7)	92.6 (91.4 - 93.6)
**Age range in years**			
18-29	1247	2.6 (1.8-3.7)	97.4 (96.3 - 98.2)
30-44	2287	5.9 (4.9-7.2)	94.1 (92.9 - 95.0)
45-59	1286	5.5 (4.0-7.4)	94.5 (92.5 - 96.0)
60-69	689	6.0 (3.8-9.5)	94.0 (90.1 - 96.4)
**Marital Status**			
Single	915	1.7 (1.0-2.8)	98.3 (97.0 - 99.1)
Married	3515	5.5 (4.6-6.5)	94.5 (93.5 - 95.4)
Separated/ Widowed/Divorced	1074	6.3 (4.7-8.4)	93.7 (91.7 - 95.2)
**Residence Status**			
Urban	1102	11.6 (9.2-14.5)	88.4 (85.9 - 90.6)
Rural	4407	2.7 (2.2-3.3)	97.3 (96.6 - 97.9)
**Socio-Economic Characteristics**			
**Education Level**			
No formal education	1988	3.0 (2.2-3.9)	97.0 (95.9 - 98.1)
Primary school education	2698	4.1 (3.3-5.1)	95.9 (94.9 - 96.7)
Secondary school education or higher	796	7.2 (5.4-9.5)	92.8 (90.6 - 94.5)
**Monthly income in USD**			
earning above 48	408	8.4 (5.8-12.0)	91.6 (88.4 - 93.9)
earning between 33–48	2061	5.1 (4.1-6.4)	94.9 (93.5 - 96.0)
earning less than 33	2591	3.1 (2.4-4.0)	96.9 (95.9 - 97.7)
**Employment Status**			
Unemployed	780	4.7 (3.3-6.7)	95.3 (93.4 - 96.7)
Employed	4729	4.3 (3.6-5.0)	95.7 (94.9 - 96.4)
**Behavioral Characteristics**			
**Alcohol consumption**			
Never	1079	7.5(5.9-9.3)	92.5(90.7-94)
Past drinkers	1260	4.1(3.1-5.5)	95.9(94.5-96.9)
Non-Heavy drinkers	2407	3.0(2.3-3.8)	97.0(96.2-97.6)
Heavy drinkers	163	4.3(1.7-10.3)	95.7(89.7-98.3
**Adequate fruits and vegetable consumption**			
Yes	971	7.0 (5.3-9.2)	93.0 (90.8 - 94.7)
No	2187	4.1 (3.2-5.3)	95.9 (94.8 - 96.8)
**Consumption of Processed Food**			
Sometimes	903	5.9 (4.4-7.9)	94.1 (91.9 - 95.7)
Rarely	724	4.5 (3.1-6.5)	95.5 (93.7 - 96.8)
Never	3834	3.9 (3.2-4.7)	96.1 (95.3 - 96.8)
**Physical inactivity**			
No	5190	4.2 (3.6-4.9)	95.8 (95.0 - 96.4)
Yes	291	6.8 (4.3-10.5)	93.2 (89.2 - 95.8)
**Tobacco Use**			
Never Smoked	4311	4.7(4.0-5.5)	95.3(94.4-96)
Past Smokers	697	3.7(2.4-5.8)	96.3(94.2-97.6)
Current Smokers	501	0.8(0.3-2)	99.2(98.0-99.7)
**Metabolic Characteristics**			
Diabetes			
No	4933	4.1 (3.5-4.8)	95.9 (95.2 - 96.5)
Yes	142	17.7 (10.7-28.0)	82.3 (70.9 - 89.9)
Hypertension			
No	4,288	3.6(2.9-4.3)	96.4(95.7-97.1)
Yes	1,219	8.2(6.4-10.3)	91.8(89.6-93.6)
Elevated Total Cholesterol			
No	5052	4.2(3.6-4.9)	95.8(95.1-96.4)
Yes	157	18.2(12.0-26.7)	81.8(73.3-88.0)

Notes: Adequate fruits and vegetable consumption is the intake of five or more servings of fruits/vegetables per day. Physical inactivity is the physical activity of less than 600 metabolic equivalent of task (MET) minutes per week.

Regarding socio-economic factors, individuals with secondary or higher level of education exhibited a higher prevalence of obesity (7.2%) compared to those with no formal education (3.0%). Income disparities were also evident, with individuals earning more than 48 USD per month showing the highest obesity prevalence (8.4%), while those earning less than 33 USD per month had a lower prevalence (3.1%). Employment status showed minimal variation, with unemployed individuals exhibiting a slightly higher prevalence of obesity (4.7%) compared to employed individuals (4.3%).

Behavioral factors were also associated with variations in obesity prevalence. Non-drinkers exhibited the highest prevalence of obesity (7.5%), while non-heavy drinkers showed a lower prevalence (3.0%). Individuals who regularly consumed daily five serving of fruits and vegetables had a higher obesity prevalence (7.0%) compared to non-consumers (4.1%). Those who sometimes consumed processed foods exhibited a higher prevalence of obesity (5.9%), whereas individuals who never consumed processed foods had a lower prevalence (3.9%). Physical inactivity was associated with a higher prevalence of obesity (6.8%) compared to those who were physically active (4.2%). Tobacco use showed a distinct pattern, with current smokers exhibiting the lowest prevalence of obesity (0.8%) compared to never-smokers (4.7%), while past smokers had a prevalence of 3.7%.

Metabolic factors were associated with higher obesity prevalence. Individuals with diabetes had a prevalence of obesity of 17.7%, compared to 4.1% among those without the condition. Similarly, individuals with hypertension exhibited a higher obesity prevalence (8.2%) compared to those without it. Elevated total cholesterol was also associated with a higher prevalence of obesity (18.2%) compared to individuals without elevated cholesterol.

The analysis of crude prevalence ratios and adjusted prevalence ratios (models 1 and 2) in Table 2 reveals several key factors related to obesity. Gender remained a strong and consistent predictor across all models with women exhibiting substantially higher obesity prevalence compared to men (APR: 4.2, 95% CI: 2.1–8.5) in model 2. Age remained an important factor associated with obesity prevalence. In Model 2, adults aged 60–69 years had a significantly higher prevalence of obesity compared to those aged 18–29 years (APR: 2.7, 95% CI: 1.03–6.9). Furthermore, rural residence was associated with lower prevalence of obesity compared to urban residence (APR: 0.3, 95% CI: 0.2–0.5),

**Table 2:** Crude, age–sex adjusted, and fully adjusted prevalence ratios (PR) of obesity by demographic, socio-economic, behavioral, and metabolic characteristics.

Variables	Crude PR	Model 1: Adjusted for Age and gender	Model 2: Fully Adjusted PR
**Demographics Characteristics**			
**Gender**			
Male	**reference**	**reference**	**reference**
Female	5.3 (3.7-7.6)	5.2(3.7-7.5)	4.2(2.1-8.5)
**Age range in years**			
18-29	**reference**	**reference**	**reference**
30-44	2.3 (1.6-3.5)	2.3 (1.5-3.4)	1.2 (0.6-2.3)
45-59	2.2 (1.3-3.5)	2.0 (1.2-3.2)	1.1 (0.5-2.8)
60-69	2.4 (1.2-4.6)	2.3 (1.2-4.4)	2.7 (1.03-6.9)
**Marital Status**			
Single	**reference**	**reference**	**reference**
Married	3.3 (1.8-6.3)	2.3(1.1-4.6)	2.2 (0.7-6.3)
Separated/ Widowed/Divorced	3.8 (2.0-7.3)	1.6 (0.8-3.4)	1.7 (0.5-5.6)
**Residence Status**			
Urban	**reference**		**reference**
Rural	0.2 (0.2-0.3)	0.2 (0.16-0.3)	0.3 (0.2-0.5)
**Socio-Economic Characteristics**			
**Education Level**			
No formal education	**reference**	**reference**	**reference**
Primary school education	1.4 (0.9-2.0)	1.7 (1.2-2.6)	1.7 (1.02-3)
Secondary school education or higher	2.4 (1.6-3.6)	3.6 (2.3-5.5)	1.7 (0.9-3.4)
**Monthly income in USD**			
earning above 48	**reference**		**reference**
earning between 33–48	0.6 (0.4-0.9)	0.6 (0.4-0.9)	0.7 (0.4-1.1)
earning less than 33	0.4 (0.2-0.6)	0.4 (0.2-0.6)	0.5 (0.3-0.9)
**Employment Status**			
Unemployed	**reference**		
Employed	1.1(0.7-1.8)	–	–
**Behavioral Characteristics**			
**Alcohol consumption**			
Never	**reference**	**reference**	**reference**
Past drinkers	0.5(0.4-0.8)	0.5 (0.3-0.7)	0.5(0.3-0.9)
Non-heavy drinkers	0.4(0.3-0.5)	0.5 (0.3-0.7)	0.5(0.3-0.9)
Heavy drinkers	0.6(0.2-1.4)	0.8 (0.3-1.9)	0.5(0.2-1.3)
**Adequate Fruits and Vegetable consumption**			
Yes	**reference**		**reference**
No	0.6 (0.4-0.8)	0.7 (0.5-0.99)	0.8 (0.5-1.3)
**Consumption of Processed Food**			
Sometimes	**reference**	**reference**	**reference**
Rarely	0.8 (0.5-1.2)	0.8 (0.5-1.2)	0.7 (0.4-1.4)
Never	0.7 (0.5-0.9)	0.6 (0.4-0.8)	0.7(0.4-1.1)
**Physical inactivity**			
No	**reference**		
Yes	1.6 (1.0-2.6)	–	–
**Tobacco Use**			
Never	**reference**	**reference**	**reference**
Past smokers	0.8(0.5-1.2)	0.7 (0.4-1.3)	1.1(0.6-1.9)
Current smokers	0.2(0.1-0.4)	0.2 (0.1-0.5)	0.3(0.1-1.2)
**Metabolic Characteristics**			
**Diabetes**			
No	**reference**	**reference**	**reference**
Yes	4.3 (2.5-7.6)	3.7 (2.2-6.3)	1.8 (0.7-4.6)
**Hypertension**			
No	**reference**		**reference**
Yes	2.3(1.7-3.1)	1.9 (1.5-2.6)	1.7 (1.1-2.5)
**Elevated Total Cholesterol**			
No	**reference**	**reference**	**reference**
Yes	4.3(2.9-6.6)	3.6 (2.4-5.2)	1.7(0.9-3.1)

Notes: Adequate fruits and vegetable consumption is the intake of five or more servings of fruits/vegetables per day. Physical inactivity is the physical activity of less than 600 metabolic equivalent of task (MET) minutes per week.

Regarding socioeconomic factors; education level was associated with obesity prevalence in all models, with higher prevalence observed among individuals with primary education compared to those with no formal education in Model 2 (APR: 1.7, 95% CI: 1.02–3). Moreover, individuals earning less than USD 33 per month exhibited lower prevalence of obesity compared to those in the highest income category (APR: 0.5, 95% CI: 0.3–0.9).

Among behavioral factors, alcohol consumption was associated with obesity prevalence in both models, with past drinkers and non-heavy drinkers showing lower obesity prevalence of obesity (APR: 0.5, 95% CI: 0.3–0.9) and (APR: 0.5, 95% CI: 0.3–0.9) respectively. However, adequate fruits and vegetable consumption, tobacco use and processed food intake associations observed in Model 1 were not retained after further adjustment in Model 2.

Among metabolic factors, individuals with hypertension had a higher prevalence of obesity compared to those without hypertension (APR: 1.7, 95% CI: 1.1–2.5) in model 2. In contrast, diabetes and elevated total cholesterol did not remain statistically significant after adjustment in Model 2.

## Discussion

The findings from the 2022 NCD STEPS Survey highlights significant associations between obesity and various demographic, socio-economic, behavioral, and metabolic factors in Rwanda. Gender, age, residence, education, income, alcohol consumption, and elevated blood pressure emerged as important predictors of obesity.

Gender was significantly associated with obesity, with women showing a markedly higher prevalence compared to men. This finding aligns with previous studies in sub-Saharan Africa, where women—particularly those in urban areas—experience higher obesity rates due to a combination of socio-cultural, biological, and lifestyle factors [13–15]. Socio-cultural norms in many African settings often associate higher body weight with wealth and health, reinforcing the acceptance of obesity among women [16,17]. Biological changes related to pregnancy, menopause, and hormonal fluctuations also contribute to women’s increased risk of obesity [13]. These findings underscore the importance of gender-sensitive interventions. In Rwanda, community-based women’s wellness groups promoting weight management through organized physical activities (such as aerobics and yoga) and nutrition workshops targeting post-menopausal women could be effective [18].

Age was also significantly associated with obesity, with older adults exhibiting a higher prevalence compared to younger individuals. This finding is consistent with previous studies conducted in Rwanda and other sub-Saharan African countries, which have shown that advancing age is linked to an increased risk of obesity [10,14,19]. Physiological factors such as a reduced basal metabolic rate, hormonal changes associated with aging, declines in physical activity, and dietary shifts toward energy-dense foods contribute to this trend [20].

Urban–rural disparities in obesity prevalence were clearly observed in this study, with urban residents showing significantly higher rates of obesity compared to rural residents. This finding is consistent with other studies in sub-Saharan Africa and globally, where urbanization has been associated with increased access to processed foods, reduced physical activity, and changes in dietary habits favoring energy-dense foods [14,15,19]..Recent studies from Rwanda and other east-african countries have similarly reported higher obesity rates among urban populations compared to rural populations, underscoring the influence of lifestyle transitions and socioeconomic changes associated with urban living [19,21,22]. Evidences showed that differences in wealth status, education level, and media exposure largely explained the rural–urban gaps in overweight and obesity Furthermore, rural residents may have greater access to unprocessed foods and engage in more physically demanding activities, which could offer protection against obesity [15,23]. However, some emerging evidence points to a narrowing of these rural–urban differences over time, especially as rural areas increasingly face challenges such as poor access to healthy foods and limited opportunities for recreational physical activity [23,24].

Higher educational attainment was significantly associated with increased obesity prevalence in this study. This finding is consistent with previous studies across Rwanda and sub-Saharan Africa, where higher education has been linked to greater obesity risk [7]. One explanation is that higher education often correlates with more sedentary occupations and increased access to calorie-dense foods, particularly in urban environments [7,24]. For instance, a study in Nigeria reported that women with secondary or higher education levels were more likely to be overweight or obese compared to those with no formal education [25]. However, findings are mixed globally; while higher education is associated with increased obesity in low- and middle-income countries, it often serves as a protective factor against obesity in high-income settings [7,23,26].

Household income was significantly associated with obesity, with individuals from lower-income households exhibiting a lower prevalence of obesity compared to those from higher-income groups [27]. This pattern is consistent with studies from Rwanda and other african countries, where higher-income individuals tend to adopt more sedentary lifestyles and consume more processed foods, contributing to obesity [7,28]. Previous research also suggests that socioeconomic transitions linked to urbanization increase exposure to obesogenic environments, disproportionately affecting higher-income groups in early stages of economic development [29,30]. Nevertheless, income effects on obesity are complex and may vary by setting, as lower-income groups in urban environments can also face higher risks due to limited access to healthy foods and safe recreational spaces [28,30].

Alcohol consumption exhibited a relationship with obesity prevalence in this study. Non-heavy drinkers and past drinkers had lower prevalence of obesity compared to those who have never drunk. These findings suggest that moderate alcohol consumers had a lower obesity prevalence, consistent with evidence from both African and Western populations [31,32]. However, the findings on the effects of alcohol on weight gain have been inconsistent and they stem from numerous confounding factors - including gender, drinking patterns, beverage type, physical activity, sleep quality, eating behaviors, and individual predisposition to weight gain. Failing to account for these variables leads to biased results, as there’s substantial variation between individuals in how alcohol affects body weight. [33–37]. In this study, given that alcohol consumption was self-reported, the potential for recall and social desirability bias should be acknowledged, as underreporting of heavy drinking is common. Further longitudinal studies are warranted to clarify causal relationships between alcohol consumption and obesity risk.

Hypertension was significantly associated with obesity in this study. Participants with elevated blood pressure exhibited a higher prevalence of obesity compared to those with normal blood pressure. This association is consistent with prior findings in Rwanda and in the region where obesity has been recognized as a major modifiable risk factor for hypertension [38–41]. Biological mechanisms underlying this relationship include increased sympathetic nervous system activation, activation of the renin-angiotensin-aldosterone system, endothelial dysfunction, and impaired renal sodium handling, all of which contribute to elevated blood pressure in individuals with excess adiposity [42–45]. Moreover, inflammation and insulin resistance associated with obesity further exacerbate blood pressure dysregulation [46,47].

Physical inactivity and processed food consumption initially appeared to be associated with obesity in the crude analysis; however, these associations did not remain statistically significant after adjustment. This suggests that their relationship with obesity in this population may be mediated by other confounding factors. Previous research has similarly shown that while lack of physical activity and unhealthy diets are important contributors to obesity [7], their independent effects may attenuate after adjusting for sociodemographic and metabolic factors. Urbanization shifts toward sedentary lifestyles, and increased consumption of energy-dense processed foods are widely recognized drivers of obesity, particularly in low- and middle-income countries [30]. While the attenuation of these associations after adjustment may reflect complex confounding or mediation pathways, it does not negate the well-established biological mechanisms linking these modifiable risk factors to obesity, nor their importance as targets for prevention strategies.

## Recommendations, future research, strengths and limitation

### Recommendations

This study’s findings offer critical insights for strengthening obesity prevention and control efforts in Rwanda. Through the National Non-Communicable Disease Strategic Plan 2020–2025 [15], the government has already implemented initiatives such as annual community NCD checks, Car-Free Days, and public awareness campaigns. While these programs are valuable, the present study highlights specific demographic, socioeconomic, and metabolic groups at higher risk—particularly women, older adults, urban residents, and individuals with elevated blood pressure.

In light of these findings, future public health strategies could benefit from tailored, community-based initiatives that promote physical activity and healthy eating among these high-risk groups. Examples include women’s wellness programs, workplace-based fitness campaigns, and urban gardening projects to enhance access to fresh produce. These recommendations are proposed as policy directions for future implementation, rather than as conclusions directly tested in this study. Their feasibility at scale will depend on resource availability, sustained behavioral engagement, and cross-sector collaboration.

### Strengths and limitations

This study’s nationally representative sample strengthens the generalizability of its findings to the adult Rwandan population. The use of adjusted prevalence ratios provides a clear understanding of the independent effects of key predictors on obesity, enhancing the study’s methodological robustness. However, the cross-sectional design limits causal inference, and the reliance on self-reported data for behavioral factors such as alcohol consumption, tobacco use, physical activity, and income may introduce recall and social desirability biases. Additionally, genetic predispositions and psychosocial influences on obesity were not assessed and warrant exploration in future research. Finally, although BMI is widely used, it does not distinguish between fat mass and lean mass, potentially limiting the precision of obesity classification.

### Future perspectives

Future studies should adopt longitudinal designs to explore the long-term effects of demographic, socioeconomic, behavioral, and metabolic factors on obesity risk. Further investigation of urban–rural differences in dietary patterns and access to healthy foods will help refine public health interventions. Evaluating the effectiveness and scalability of existing national initiatives, such as Car-Free Days and NCD community screenings, will be critical for strengthening Rwanda’s obesity prevention strategies. Research should also consider incorporating more comprehensive body composition measures and investigating the impact of psychosocial factors on obesity development.

## Conclusion

This study provides the first peer-reviewed adjusted analysis of the nationally representative 2022 Rwanda NCD STEPS dataset, offering new evidence on the independent sociodemographic, behavioral, and metabolic predictors of obesity in Rwanda. Gender, age, residence, education, income, alcohol consumption, and elevated blood pressure emerged as key predictors, emphasizing the need for targeted public health interventions. Women and older adults, particularly in urban areas, are at higher risk, underscoring the importance of gender- and age-specific strategies. Addressing these factors through comprehensive programs focused on improving health literacy, promoting physical activity, and managing metabolic conditions like hypertension will be essential in curbing obesity in Rwanda. Future research should further investigate these relationships over time and evaluate the effectiveness of ongoing prevention efforts.
